# Dimensional analysis meets AI for non-Newtonian droplet generation†

**DOI:** 10.1039/d4lc00946k

**Published:** 2025-02-12

**Authors:** Farnoosh Hormozinezhad, Claire Barnes, Alexandre Fabregat, Salvatore Cito, Francesco Del Giudice

**Affiliations:** a Departament d'Enginyeria Mecanica, Universitat Rovira i Virgili Tarragona Spain; b Department of Biomedical Engineering, Swansea University UK; c Complex Fluids Research Group, Department of Chemical Engineering, Swansea University Fabian Way SA1 8EN Swansea UK francesco.delgiudice@swansea.ac.uk

## Abstract

Non-Newtonian droplets are used across various applications, including pharmaceuticals, food processing, drug delivery and material science. However, predicting droplet formation using such complex fluids is challenging due to the intricate multiphase interactions between fluids with varying viscosities, elastic properties and geometrical constraints. In this study, we introduce a novel hybrid machine-learning architecture that integrates dimensional analysis with machine learning to predict the flow rates required to generate droplets with specified sizes in systems involving non-Newtonian fluids. Unlike previous approaches, our model is designed to accommodate shear-rate-dependent viscosities and a simple estimate of the elastic properties of the fluids. It provides accurate predictions of the dispersed and continuous phases flow rates for given droplet length, height, and viscosity curves, even when the fluid properties deviate from those used during training. Our model demonstrates strong predictive power, achieving *R*^2^ values of up to 0.82 for unseen data. The significance of our work lies in its ability to generalize across a broad range of non-Newtonian systems having different viscosity curves, offering a powerful tool for optimizing droplet generation. This model represents a significant advancement in the application of machine learning to microfluidics, providing new opportunities for efficient experimental design in complex multiphase systems.

## Abstract 2

We introduce a novel hybrid machine-learning architecture that integrates dimensional analysis with machine learning to predict the flow rates required to generate droplets with specified sizes in systems involving non-Newtonian fluids.

## Graphical abstract

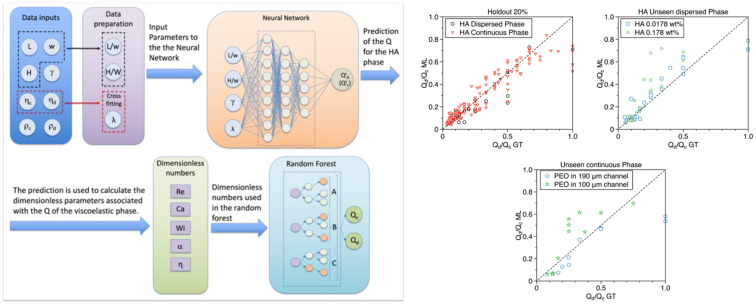

## Introduction

Non-Newtonian fluids exhibit both elastic and viscous behavior, with flow characteristics that deviate from Newton's law, *τ* = *ηγ̇*, where *τ* is the shear stress, *η* is the shear viscosity, and *γ̇* is the shear rate.^1^ A key feature of these fluids is their shear-rate-dependent viscosity, leading to behaviors such as shear-thinning or shear-thickening.^1^ Non-Newtonian fluids are ubiquitous in everyday products like toothpaste, creams, detergents, and paints, making them equally prevalent in industrial processes. Non-Newtonian liquids are often encountered in the form of droplets,^2–4^ generated by interactions with a non-miscible phase, typically oil. Droplet formation involving non-Newtonian fluids is relevant to a wide range of applications, including enhanced oil recovery, renewable fuels, fluidized bed reactors,^5^ and in the formulation of cosmetics,^6^ drug delivery systems,^7^ microgel synthesis,^8^ and encapsulation technologies.^9,10^ Despite their importance, understanding the mechanisms behind droplet formation, even in simpler Newtonian cases, remains challenging. Predicting the experimental conditions required to generate droplets of a specific size before conducting experiments is often unattainable.

While there is extensive research on droplet microfluidics involving Newtonian liquids,^2,3,11^ far fewer studies address the complexities of non-Newtonian fluids. Existing work has focused on linking droplet size to experimental parameters^12–26^ or analyzing the breakup dynamics of viscoelastic filaments during droplet formation,^27–30^ often considered a hallmark of viscoelasticity. A key takeaway from these studies is the absence of a single master curve or universal theory to describe droplet formation in non-Newtonian fluids, even for specific geometries like flow-focusing or T-junctions. Even attempts at using dimensional analysis,^31^ where dimensionless parameters are employed to describe the relevant forces taking place in the droplet microfluidic processes, have not been successful at providing a single master curve that can be used to design droplet microfluidic experiments appropriately. This gap also persists for Newtonian droplet formation, which has led to the use of machine learning tools to predict the design and experimental conditions for generating droplets.^32^ For example, Lashkaripour *et al.*^33,34^ developed machine learning models to predict the microfluidic design and experimental conditions necessary to produce droplets of a specified size, while Chagot *et al.*^35^ used machine learning to predict droplet generation conditions in the presence of various surfactants. Other studies have similarly leveraged machine learning to predict experimental conditions for droplet formation in microfluidic systems.^36–42^ However, these models are typically limited to Newtonian fluids with constant viscosity and fail to account for the rheological behaviour of more complex fluids, restricting their broader applicability.

In this study, we introduce a novel hybrid machine-learning architecture that integrates dimensional analysis with machine learning to predict the flow rates required for generating droplets of specified sizes in systems with non-Newtonian fluids. It accurately predicts the flow rates *Q*_d_ and *Q*_c_ needed for given droplet dimensions and fluid properties, even when the viscosity curves deviate from those used in training. Our model demonstrates strong predictive power, achieving *R*^2^ values of up to 0.82 on unseen data and maintains robustness across different microfluidic geometries. The broader significance of our work lies in its ability to generalize across a wide range of non-Newtonian fluid systems, providing a powerful tool for optimizing droplet generation. This model represents a significant advance in the application of machine learning to microfluidics, paving the way for more efficient experimental design in complex multiphase systems.

## Theoretical background

We provide a brief background about dimensional analysis theory and its application to droplet microfluidics problems.

## Dimensional analysis

Dimensional analysis is a powerful mathematical tool used to simplify complex physical problems by reducing the number of variables needed to describe the system.^31^ By leveraging fundamental physical quantities like length, mass, and time, it allows for the transformation of a large number of parameters into a smaller set of dimensionless numbers that capture the essence of the problem's physics. This reduction provides a more manageable framework to analyze and interpret experimental data and theoretical predictions. A classic application of dimensional analysis in engineering is pipe flow,^43^ where it is possible to reduce the full characterization of the system to just three dimensionless parameters: the Reynolds number, the relative roughness, and the friction factor. These parameters allow for a comprehensive description of how fluid flows through a pipe, encompassing both the fluid's viscosity and the roughness of the pipe's inner surface, without needing to consider every individual physical property directly.

The foundation of dimensional analysis lies in the Buckingham Pi theorem,^31^ which provides a systematic approach to determine the number of dimensionless parameters, but it does not yield the specific functional relationship between them. For this reason, experiments or simulations are required to explore and establish these relationships. According to the Buckingham theorem, for a system with *N* variables and *D* independent physical dimensions (such as length, mass, and time), the number of dimensionless groups is given by *G* = *N* − *D*. For example, in the case of a Newtonian liquid flowing through a pipe, we consider variables like the fluid density *ρ*, viscosity *η*, velocity *V*, pipe diameter *D*, and pipe roughness *ε*, which generate a wall shear stress *τ*_w_. Here, *N* = 6 parameters and *D* = 3 independent dimensions (length, mass, time), resulting in three dimensionless groups (*G* = *N* − *D* = 3) that fully describe the system, *i.e.*, the Reynolds number, the relative roughness, and the friction factor. Importantly, the construction of these dimensionless numbers is not unique, and while guidelines exist to aid in their generation,^43^ no absolute recipe governs their selection. Thus, the specific dimensionless quantities may vary depending on the approach taken or the focus of the analysis.

## Dimensionless numbers in droplet microfluidics

**Fig. 1:**
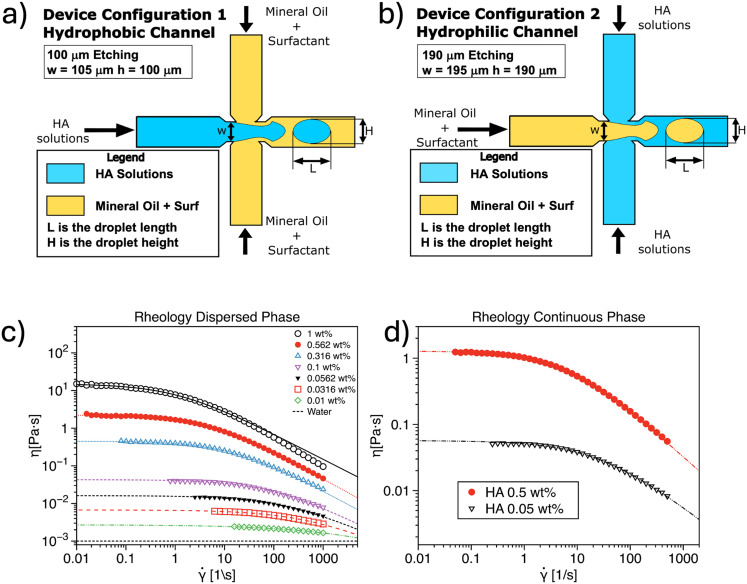
Schematic representation of the flow focusing microfluidic devices employed to generate droplets using a) hyaluronic acid (HA) aqueous solutions as the dispersed phase and b) HA aqueous solutions a the continuous phase. Dimensions are not to scale. c) Viscosity curve as a function of the shear rate *γ̇* for the HA solutions employed in the microfluidic device in (a). d) Same as (c) for the microfluidic device in (b). The dashed lines in (b and c) is the cross model fit (refer to the text for more details).

We focus on droplet microfluidics, applying dimensional analysis to our specific system. Here, two immiscible liquids, a dispersed phase and a continuous phase, meet at the junction of a microfluidic device to generate droplets (Fig. 1(a and b)). For instance, the dispersed phase can be an aqueous solution while the continuous phase can be an oil phase (Fig. 1a) or *vice versa* (Fig. 1b). The droplets generated will have a characteristic length *L* and height *H*. Both the dispersed and continuous phases flowing with flow rates *Q*_d_ and *Q*_c_, respectively, have their own density and viscosity, denoted as *ρ*_d_, *η*_d_ for the dispersed phase and *ρ*_c_, *η*_c_ for the continuous phase. In our experiments, one of these phases (but not both) exhibits shear-thinning behaviour, where the viscosity decreases with increasing shear rate.^1^ Additionally, one of the phases will display non-Newtonian elastic properties, quantified by its longest relaxation time *λ*. At any given time, only one of the two phases, either the continuous or the dispersed phase, exhibits viscoelasticity, but not both. Since the two fluids are immiscible, they possess an interfacial tension *γ*. The microfluidic device also has a junction width *w* and a etching height *h*.

Given this system, there are *N* = 12 parameters and *D* = 3 independent dimensions (length, mass, time), which means that the problem can be described by *G* = *N* − *D* = 12–3 = 9 dimensionless parameters. Based on previous studies in droplet microfluidics,^2–4^ we have identified the following dimensionless parameters that will be used to describe the system.

The Reynolds number, one for the dispersed and one for the continuous phase, represents the ratio of inertial forces to viscous forces:1
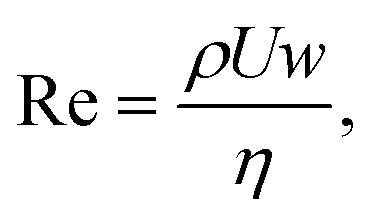
where *ρ* is the fluid density, *η* is the fluid viscosity, *U* is the fluid velocity, and *w* is the device width (Fig. 1a).

The capillary number, one for the dispersed and one for the continuous phase represents the ratio of viscous forces to interfacial forces:2
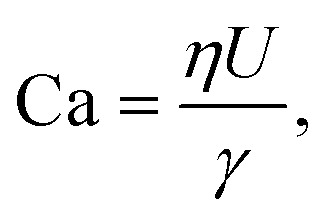
where *γ* is the interfacial tension.

The Weissenberg number quantifies the ratio of elastic to viscous forces:3
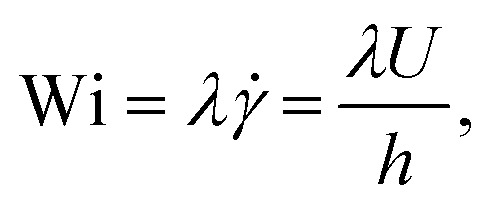
where *λ* is the longest relaxation time and *h* is the etching depth of the device.

The normalized droplet length and height are given as:4
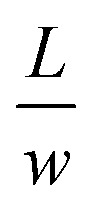
5
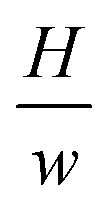
The viscosity ratio is defined as:6
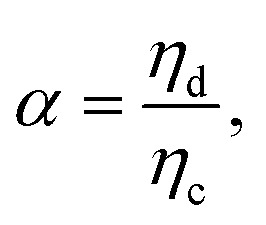
where the subscripts d and c refer to the dispersed and continuous phase, respectively.

Finally, the flow rate ratio is:7
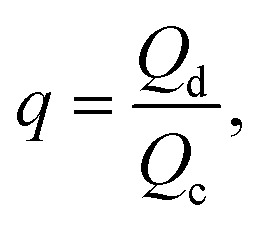
where the symbol *Q* represents the volumetric flow rate. Taken together the 9 dimensionless parameters can be used to describe droplet generation experiments when one of the two phases is non-Newtonian. Please note that, for shear-thinning liquids, we used the actual viscosity values at the average shear rate in the channel, eliminating the need to separately account for the zero-shear viscosity and the degree of shear-thinning. Specifically, each dimensionless parameter above is calculated using the viscosity at the shear rate *γ̇* = *U*/*h*, where *h* is the etching depth, ensuring that we accurately computed the viscosity to reflects the flow conditions within the channel.

## Materials and methods

### Sample preparation and characterization

We employed aqueous solutions of hyaluronic acid (HA, Sigma Aldrich, UK) at different mass concentrations, as well as mineral oil (Sigma Aldrich, UK) with varying concentrations and types of surfactants, depending on the device configuration. When the non-Newtonian liquid was in the dispersed phase (Fig. 1a), we prepared HA solutions with concentrations ranging from 1 wt% to 0.01 wt% to capture varying degrees of elasticity and shear-thinning. The surfactant type (Span 80) and concentration in the oil phase was held constant at 1 wt%, following the conditions established in previous studies.^9^ When the non-Newtonian liquid was used as the continuous phase (Fig. 1b), two HA solutions were selected, specifically 0.5 wt% and 0.05 wt%, while the dispersed phase consisted of oil with either Tween 20 or Span 80 at different concentrations, spanning a range of interfacial tension values. The aqueous solutions were prepared by directly adding polymer powder to water, followed by overnight stirring at room temperature using a magnetic stirrer to ensure full dissolution. Dilutions from the stock solution were used to achieve the desired concentrations. For the oil phase, liquid surfactant was added directly to mineral oil and stirred to achieve homogeneity. From previous work,^9^ we observed that low HA concentrations had little effect on the interfacial tension between the mineral oil and Span 80 at 1 wt%. Consequently, when the non-Newtonian phase was used as the dispersed phase, we adopted the interfacial tension value of 3.63 mN m, in agreement with our previous work.^9^ When the non-Newtonian phase was employed as the continuous phase, interfacial tension was measured using a force tensiometer (Sigma 702, Biolin Scientific) equipped with a du Nouy ring. In this process, the lighter phase was poured on top of the ring after immersion in the heavier phase. The force required to break the ring from the interface was recorded using the device's built-in microbalance and was used to calculate the interfacial tension. In agreement with the observation^9^ that the HA concentration did not significantly affect the interfacial tension *γ*, we measured the interfacial tension between water and mineral oil containing various types and concentrations of surfactants, with results reported in Table 1.

**Table 1:** Interfacial tension values for water and mineral oil at different surfactant types and concentrations

Surfactant type and concentration	Interfacial tension [mN m]
Span 80–0 wt%	25.06 ± 0.16
Span 80–0.03 wt%	9.07 ± 0.12
Span 80–0.3 wt%	4.51 ± 0.16
Span 80–1 wt%	3.91 ± 0.1
Tween 20–0.03 wt%	6.94 ± 0.48
Tween 20–0.3 wt%	2.49 ± 0.2
Tween 20–1 wt%	1.83 ± 0.02

The rheological characterization of the solutions was performed using an Anton Paar MCR702 rotational rheometer, equipped with a cone and plate geometry (50 mm diameter, 1° angle), and measurements were conducted at 20 °C (Fig. 1c and d). The viscosity curves showed a constant viscosity at low shear rates, followed by a shear-thinning region, with the degree of shear-thinning depending on HA concentration, as expected based on previous literature.^44,45^ Minor discrepancies between viscosity curves in Fig. 1a and b are attributed to different operators preparing the solutions.

The viscosity curves were fitted using the cross model,^1^ assuming an infinite viscosity *η*_∞_ = 0:8
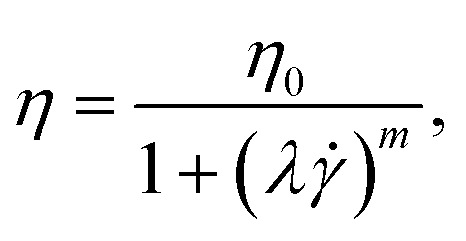
where *λ* represents the characteristic relaxation time used to estimate the longest relaxation time, *η*_0_ is the zero-shear viscosity, and *m* is a parameter that characterizes the strength of shear-thinning. We recognise that estimating *λ via* the cross-model is a simplification compared to performing small angle oscillatory shear measurements. However, this approach has also been previously employed in the literature^45–47^ and it is a valid approach to quantify the elasticity of a fluid *via* a simple viscosity curve measurement.

### Experimental setup

The experimental setup used in this study is similar to the one employed in our previous work,^36^ and is summarized below. For the experiments where the non-Newtonian phase was in the dispersed phase, we used a commercial glass microfluidic device with an etching depth of 100 μm (*h* = 100 μm and *w* = 105 μm) and a hydrophobic coating (Dolomite Microfluidics, UK). In cases where the non-Newtonian phase was in the continuous phase, we utilized a glass microfluidic device with an etching depth of 190 μm (*h* = 190 μm and *w* = 195 μm) without any surface coating (Dolomite Microfluidics, UK). This distinction is crucial for meeting the surface wetting requirements necessary to generate either aqueous or oil droplets, as discussed in previous studies.^48^

Flow rates were controlled using a syringe pump equipped with two independent flow units and pressure sensors on each flow stream (Dolomite Quad Pumps). The system was fitted with Mitos Quad Pump Green syringe pairs (Dolomite Microfluidics), providing a flow rate range from 5 μl min^−1^ to 1250 μl min^−1^. We monitored the overall pressure in the microfluidic channels using the embedded pressure sensors, allowing us to track pressure drop fluctuations throughout the device. After setting the desired flow rate, we waited for the flow to stabilize, defined by the point when pressure fluctuations around the mean pressure drop remained consistently within 10% of the overall value. Notably, larger fluctuations were observed at higher polymer concentrations.

The flow within the microfluidic channel was visualized using an inverted microscope (Zeiss Primovert) coupled with a high-speed camera (Photron Mini UX-50). The camera operated at frame rates ranging from 100 fps to 2000 fps, depending on the requirements of the experiment. All videos were analyzed using an in-house MATLAB code to extract the relevant parameters.

## Results and discussion

We first present the results from the experiments and compare them with the literature to gain confidence about their reliability. We also show that a simplistic application of the dimensional analysis considering the dimensionless parameters as independent is insufficient to generate a single master curve for the experimental data. For this reason, we then introduce a hybrid machine-learning architecture that can predict the experimental values of the flow rates required to generate droplets of a given size, given specific fluid properties of either the continuous or the dispersed phase. We test the accuracy of the hybrid machine-learning architecture on both holdout data from training data, and completely unseen data from fluids having viscosity curves that were not employed in the training.

### Non-Newtonian fluids in the dispersed phase

**Fig. 2:**
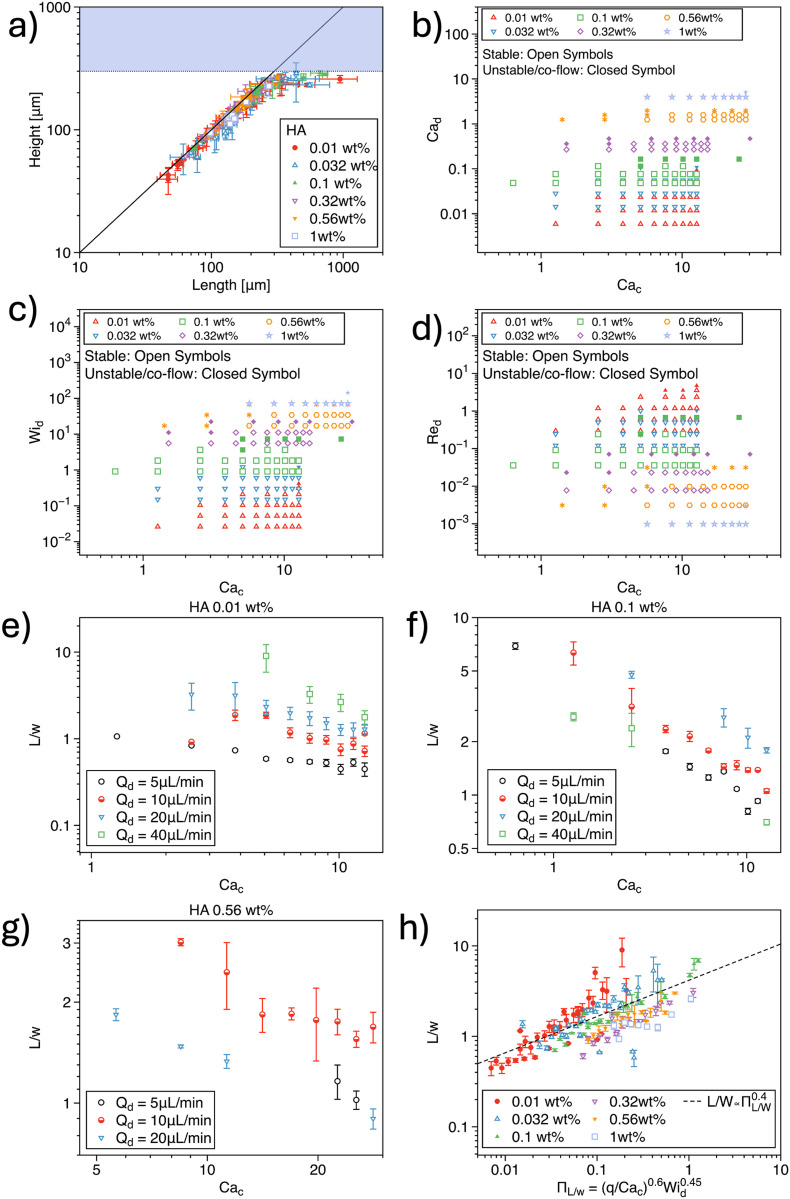
a) Droplet height *H* as a function of the droplet length *L* for several hyaluronic acid (HA) concentrations used as the dispersed phase. The coloured area is inaccessible to the droplet, being the downstream width of 290 μm. (b–d) Stability diagram for droplet formation as a function of different dimensionless numbers. (e–g) Droplet length *L* normalised by the channel width *w* as a function of the capillary number of the continuous phase Ca_c_ for different imposed volumetric flow rate and different hyaluronic acid concentrations in water. h) *L*/*w* as a function of a combination of dimensionless parameters. The data follow a common trend but do not overlap on a single mastercurve.

We begin by discussing the formation of non-Newtonian droplets using mineral oil as the continuous phase (Fig. 2). In agreement with our previous study,^36^ we plotted the droplet height *H* as a function of the droplet length *L* and observed that most droplets formed were circular in shape (Fig. 2a). The only exceptions occurred when the droplet length exceeded the height, particularly for low values of the flow rate ratio *q* or when the droplet size approached the maximum width of the expansion area, which was 290 μm for the device employed. We then proceeded to evaluate the stability of droplet formation using a combination of dimensionless numbers, namely Ca_d_, Ca_c_, Wi_d_, and Re_d_ (Fig. 2b–d). For a fixed value of Ca_c_, an increase in Ca_d_ resulted in unstable droplet formation (Fig. 2b). Similarly, for a fixed Ca_d_, larger values of Ca_c_ were required to stabilize droplet generation, particularly with increasing polymer concentration. Indeed, increasing the polymer concentration led to higher fluid viscoelasticity, represented by an increase in Wi_d_, which required higher values of Ca_c_ to maintain stable droplet formation (Fig. 2c). These results are consistent with previous works. For example, Rostami and Morini^20,21^ studied non-Newtonian droplet formation using xanthan gum as the dispersed phase and observed that droplet formation transitioned into a jetting or unstable regime as Ca_d_ increased relative to Ca_c_. This phenomenon can be explained by the fact that higher values of Ca_d_, associated with increased *Q*_d_, necessitate larger values of Ca_c_ and *Q*_c_ to generate stable droplets.

Next, we studied the behavior of normalized droplet length *L*/*w* as a function of Ca_c_ for different HA concentrations (Fig. 2e–g). Regardless of the polymer concentration—and consequently the degree of elasticity—we observed a consistent decrease in droplet size with increasing Ca_c_. This finding aligns with previous studies that reported similar observations.^20,21,23,49^

We then compiled all the data for normalized droplet length across different polymer concentrations and examined their scaling with respect to dimensionless numbers (Fig. S1†). The droplet length scaled as *L*/*w* ∝ Ca^−0.6^_c_ (Fig. S1a†), which is in reasonable agreement with the *L*/*w* ∝ Ca^−0.4^_c_ dependence found by Shahrivar *et al.*^9^ Furthermore, we observed a scaling of *L*/*w* ∝ *q*^0.6^ (Fig. S1b†), in agreement with prior studies,^9^ and *L*/*w* ∝ Wi^0.45^_d_ (Fig. S1c†). However, when plotting *L*/*w* against *α*, Ca_d_, Re_c_, and Re_d_, no meaningful overall correlation was found (Fig. S1(d–g)†). This suggests that the influence of these parameters on *L*/*w* is less significant than the impact of other variables.

Finally, we plotted *L*/*w* as a function of the combined scalings, specifically *L*/*w* ∝ (*q*/Ca_c_)^0.6^Wi^0.45^_d_, and observed that, while the data followed a common trend, no definitive master curve could be established (Fig. 2h). This does not indicate that the dimensionless numbers are inadequate for describing the system; rather, it suggests that the functional relationship governing droplet formation is more complex than the scaling laws identified in this study.

Taken together, our results show a good agreement with existing literature. However, a simple application of dimensional analysis cannot fully capture the complexity of non-Newtonian droplet formation. The lack of a clear master curve suggests that the functional dependencies among the variables are more intricate than those presented here. This also means that we could not establish any simple relationship that can guide users in identifying the values of *Q*_d_ and *Q*_c_ required to generate droplets of a given *L* and *H*.

### Non-Newtonian fluids in the continuous phase

**Fig. 3:**
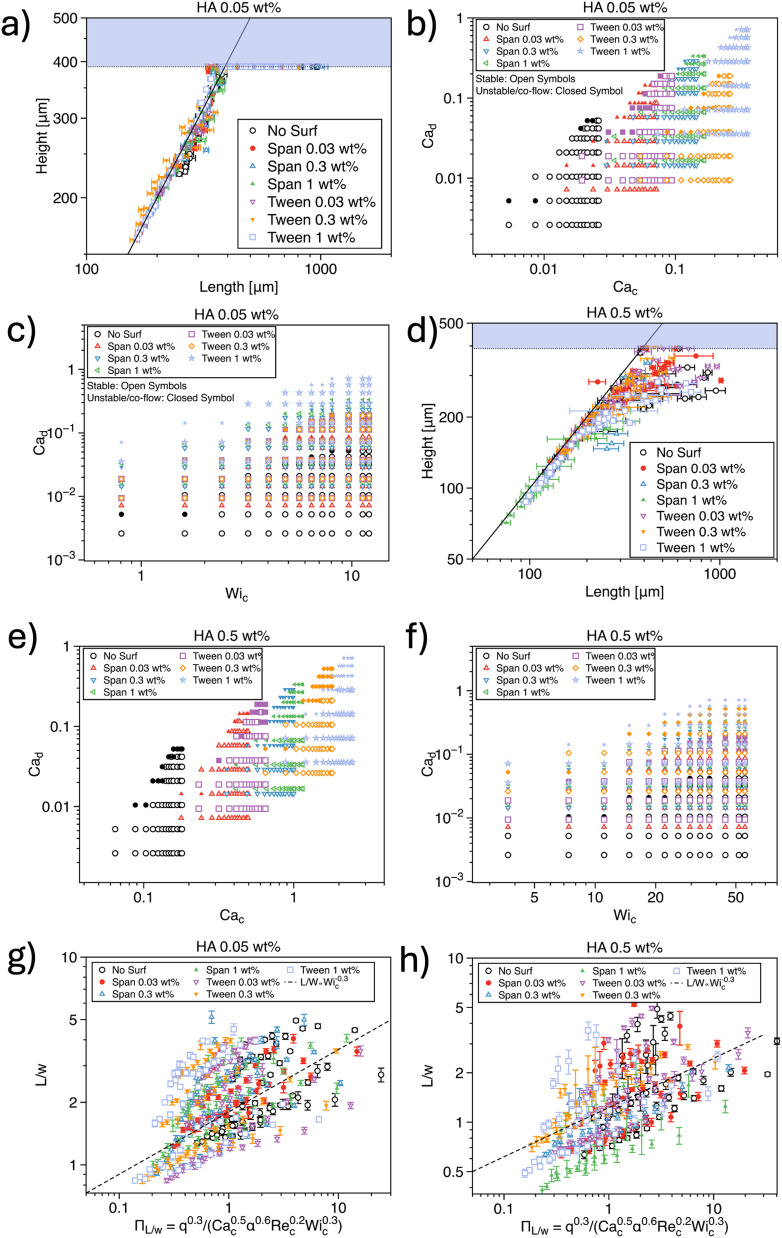
a) Droplet height *H* as a function of the droplet length *L* for hyaluronic acid (HA) concentration of 0.05 wt% used as the continuous phase. The coloured area is inaccessible to the droplet, being the downstream width of 390 μm. (b and c) Stability diagram for droplet formation as a function of different dimensionless numbers for HA 0.05 wt%. d) Same as (a) for HA 0.5 wt%. (e and f) Same as (b and c) for HA 0.5 wt% g and h) *L*/*w* as a function of a combination of dimensionless parameters for HA 0.05 wt% (g) and HA 0.5 wt% (h). The data follow a common trend but do not overlap on a single mastercurve.

We now discuss the formation of mineral oil droplets with non-Newtonian HA as the continuous phase (Fig. 3). We followed the same approach used in the study of non-Newtonian droplets surrounded by mineral oil (Fig. 2). The dynamics of droplet formation varied with the concentration of HA in the non-Newtonian fluids, leading us to organize the data according to the polymer concentration used.

Let us begin with the HA 0.05 wt% (Fig. 3(a–c)). We primarily generated circular droplets (Fig. 3a), which is consistent with our experiments using HA as the dispersed phase. From the stability diagram featuring Ca_d_ as a function of Ca_c_, we observed that, for a constant value of Ca_c_, an increase in Ca_d_ led to instability in droplet formation. This finding aligns with previous studies. For instance, Derzsi *et al.*^13^ used a non-Newtonian, near-constant viscosity fluid as a continuous phase and reported that for 10^−3^ < Ca_c_ < 10^−2^, increasing Ca_d_ resulted in a transition from droplet generation to jetting. We also explored the influence of continuous-phase viscoelasticity on droplet formation stability, finding that despite large values of Wi_c_ > 1, the droplet formation process remained predominantly stable (Fig. 3c).

We further evaluated the functional relationship between *L*/*w* and various dimensionless parameters across the entire dataset for HA 0.05 wt% in the continuous phase (Fig. S2†). Our results showed that *L*/*w* ∝ Ca^−0.5^_c_, *L*/*w* ∝ *q*^0.3^, *L*/*w* ∝ Wi^−0.3^_c_, *L*/*w* ∝ *α*^−0.6^, and *L*/*w* ∝ Re^−0.2^_c_. However, we could not clearly identify a dependency on Ca_d_. These trends are in good agreement with findings reported in the literature. For example, Chen *et al.*^50^ observed that *L*/*w* ∝ Ca^−0.454^_c_ for a flow-focusing configuration similar to the one used in our study, closely matching our observed scaling. They also reported *L*/*w* ∝ *q*^−0.41^, which aligns with our observations. Similar trends have also been documented by Derzsi *et al.*^13^

For HA 0.5 wt% in the continuous phase, the overall trends were similar to those observed for the HA 0.05 wt% data, although there were some key differences. In terms of droplet shape, while the predominant shape remained circular, we noted a larger variation in droplet size compared to the HA 0.05 wt% samples (Fig. 3d). Numerical studies by Gupta and Sbragaglia^16^ did not report a significant change in the standard deviation of droplet length, whereas experiments by Ren *et al.*^14^ using a co-flow geometry found increased variability in droplet size with shear-thinning liquids, consistent with our findings. Regarding droplet generation stability, we observed a trend similar to the HA 0.05 wt% data but with a more pronounced instability region at larger values of Ca_d_ for a fixed Ca_c_ (Fig. 3e) and Wi_c_ (Fig. 3f).

In terms of normalized droplet length as a function of individual dimensionless parameters, we observed a similar overall trend, but with slightly different scaling relationships (Fig. S3†). Specifically, we found *L*/*w* ∝ Ca^−0.84^_d_, *L*/*w* ∝ *q*^−0.54^, *L*/*w* ∝ Wi^−0.31^_c_, *L*/*w* ∝ *α*^−0.5^, and *L*/*w* ∝ Re^−0.2^_c_. The most notable deviations appeared in the scaling with Ca_d_ and *q*, though it is challenging to fully appreciate these differences when considering the data more broadly. Despite the differences in specific numerical values, the overall trends were consistent with those for HA 0.05 wt%.

We also attempted to identify a potential master curve by combining all the scaling relationships and using averaged scalings across the different datasets. However, the data exhibited significant deviations from the simple power-law scaling identified by the best power-law fit (Fig. 3(g and h)), with deviations notably larger than those observed for non-Newtonian HA in the dispersed phase.

Taken together, our results are generally in agreement with findings reported in the literature. However, a straightforward dimensional analysis, assuming the dimensionless parameters to be independent, is insufficient to produce a unified master curve. As in the previous case, we could not establish any simple relationship that can guide users in identifying the values of *Q*_d_ and *Q*_c_ required to generate droplets of a given *L* and *H*.

### Design and performance of the hybrid machine-learning architecture

**Fig. 4:**
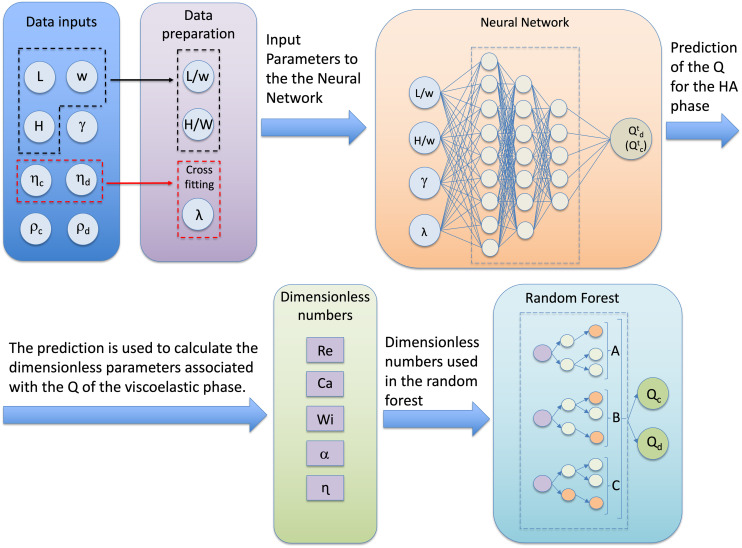
Schematic representation of the hybrid machine-learning architecture developed in this work. The users first input the variables related to desired droplet length *L* and height *H*, together with a series of fluid properties, specifically *ρ*_d_, *ρ*_c_, *η*_d_, *η*_c_, and the surface tension *γ*. Only one of the two viscosity curves is shear-rate dependent, either the continuous or the dispersed phase. The cross model is applied to estimate the longest relaxation time *λ* for the shear-rate dependent viscosity. Then, the dimensionless droplet length *L*/*w* and height *H/w* are computed and combined with *λ* and *γ* as input parameters to the first neural network where an initial prediction of the flow rate of the non-Newtonian phase *Q*^*t*^ is obtained, where the superscript *t* stands for ‘temporary variable’. The flow rate of the non-Newtonian phase *Q* is then employed with the data input to evaluate a series of dimensionless parameters that are then used, together with the viscosity curve *η*, as input parameters to the random forest algorithm to predict *Q*_d_ and *Q*_c_.

One of the key challenges in droplet microfluidics, especially with non-Newtonian fluids, is to identify the values of *Q*_d_ and *Q*_c_ required to generate droplets having a specific length *L* and height *H*. Since the simplistic dimensional analysis was unable to provide a simple generalised tool to identify such potential values of *Q*_c_ and *Q*_d_, we developed a hybrid machine-learning architecture based on dimensional analysis to predict the experimental values of *Q*_d_ and *Q*_c_ for given droplet length *L*, height *H*, and the viscosity curve of the non-Newtonian phases (Fig. 4). This approach combines the strengths of both neural networks and dimensional analysis to account for the complex interactions between parameters that the traditional approach could not capture. Under a practical point of view, the hybrid machine-learning can provide the values of *Q*_d_ and *Q*_c_ required to generate the desired droplets using the desired liquids. To the best of our knowledge, only one previous study^51^ has employed dimensional analysis in conjunction with machine learning for droplet generation in microfluidic devices, and this was limited to Newtonian fluids. In their approach, the authors used dimensionless parameters as both input and output variables, with up to 12 dimensionless inputs to predict 2 dimensionless outputs. While their method achieved good prediction accuracy, it may have been prone to overfitting,^52,53^ as it required a large number of input parameters to generate relatively few output values. Moreover, from a user perspective, their method required prior knowledge of specific dimensionless numbers to obtain predictions, which can be inconvenient or even impossible in many cases. In our study, the only known dimensionless numbers are *L*/*w* and *H/w*, which correspond to the desired droplet dimensions. However, knowing these alone is insufficient for accurate predictions, as multiple values of the flow rate ratio *q* can yield the same values of *L*/*w* and *H/w*. Additionally, in our system, one of the fluids has a shear-rate dependent viscosity, adding further complexity to the problem. It is also challenging to hypothesize a value for Ca for the non-Newtonian phase, as the viscosity is directly influenced by fluid velocity due to its shear-rate dependence. From a practical standpoint, the user typically knows the physical properties of the two liquids, such as their viscosity values, viscosity curves, densities, and interfacial tensions, along with the device width *w* and the desired droplet size values *L* and *H*. The goal is to predict the flow rates *Q*_c_ and *Q*_d_ required to produce droplets of the specified size with the given fluid combination. We designed our hybrid machine-learning with these user requirements in mind, ensuring that the system could effectively bridge the gap between known physical parameters and the experimental conditions needed for precise droplet generation.

#### Design

The hybrid machine-learning operates as follows. Users first input variables related to the desired droplet length *L* and height *H*, alongside a set of fluid properties, specifically the densities *ρ*_d_ and *ρ*_c_, viscosities *η*_d_ and *η*_c_, and the interfacial tension *γ*. It is important to note that only one of the viscosity values—either for the continuous or the dispersed phase—exhibits shear-rate dependence. For the phase with shear-rate dependent viscosity, we apply the cross model to estimate the longest relaxation time *λ*.

Once this preliminary information is provided, the model computes the dimensionless droplet length *L*/*w* and height *H/w* and combines these with *λ* and *γ* as input parameters for the first neural network. This approach is selected because neural networks excel at identifying complex, non-linear relationships within datasets, even when the number of input parameters is relatively small. This network then generates an initial prediction of the flow rate of the non-Newtonian phase, denoted as *Q*^*t*^, where the superscript stands for ‘temporary variable’. The challenge in this task lies in the fact that the same set of input parameters can result in multiple possible output combinations. To address this, we generate a temporary estimate for *Q*^*t*^ (*i.e.*, either *Q*^*t*^_c_ or *Q*^*t*^_d_) that can later inform additional model constraints. Subsequently, this predicted flow rate of the non-Newtonian phase *Q*^*t*^ is used, along with the initial input data, to calculate a series of dimensionless parameters. These parameters, together with the viscosity curve *η,* serve as input to a random forest algorithm, which refines the prediction and provides the final values of *Q*_d_ and *Q*_c_, based on the training dataset. For the training dataset, we employed the dimensionless numbers associated with the experimental data described in the previous sections. Random forests are well-suited for this task due to their ability to handle uncertainty and noise in the input data. Random forests are robust to noisy or irrelevant features, as each decision tree in the ensemble focuses on different subsets of the data. This is particularly important because the dimensionless parameters used to guide the model are based on the initial estimate of *Q* from the first neural network, which may contain uncertainty. By aggregating the results of multiple trees, random forests can ‘vote’ on the most likely outcomes for *Q*_c_ and *Q*_d_, which helps further mitigate the effect of any uncertainty in the inputs. The final output is an estimate of *Q*_c_ and *Q*_d_ that is optimized to produce the required droplet sizes based on the input parameters and fluid characteristics. The model is trained on data from a variety of viscosity curves (Fig. 1(c and d)) and a single geometry channel. This two-step process is both flexible—able to discard irrelevant information—and robust, capable of capturing the complex relationships between the fluid properties and the resulting flow rates. Overall, this hybrid neural network–random forest approach combines the strengths of both models: the neural network's ability to detect complex relationships and the random forest's robustness to noisy or uncertain input parameters.

#### Performance of the hybrid machine-learning architecture

**Fig. 5:**
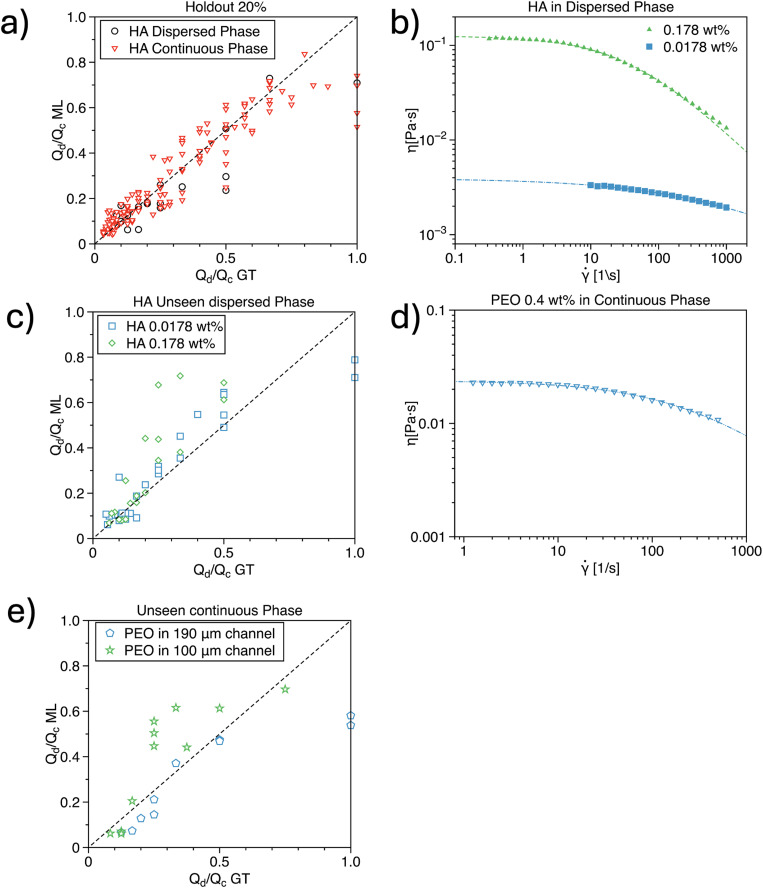
a) Flow rate ratio predicted by the hybrid network as a function of the same ratio from the ground truth holdout data, meaning 20% of the entire dataset with HA concentration as in Fig. 1(c and d). The correlation is *R*^2^ = 0.71 for HA in the dispersed phase and *R*^2^ = 0.82 for HA in the continuous phase. b) Viscosity curves for HA concentrations in the dispersed phase, unseen by the network, meaning that no training dataset was provided on those concentrations. c) Flow rate ratio predicted by the hybrid network as a function of the same ratio from the ground truth data for the unseen HA concentrations in the dispersed phase. The correlation is *R*^2^ = 0.8 for HA 0.0178 wt% and *R*^2^ = 0.46 for HA 0.178 wt%. d) Viscosity curve for a PEO concentration on the continuous phase, unseen by the network. e) Flow low rate ratio predicted by the hybrid network as a function of the same ratio from the ground truth data for PEO unseen curves in the 190 μm and 100 μm devices. The correlation is *R*^2^ = 0.83 in the 190 μm device and *R*^2^ = 0.48 in the 100 μm device.

We now proceed to describe the accuracy of the hybrid machine-learning architecture in different conditions. In agreement with previous works,^36^ we evaluated the accuracy by using the flow rate ratio rather than the independent values of the flow rate. In fact, the droplet size depends upon the ratio between the two flow rates rather than their individual value.^2^ We first present the results related to the accuracy of the hybrid machine-learning architecture with respect to the holdout data (Fig. 5a). The holdout data represent 20% of the entire experimental dataset used to train the algorithm. This test evaluates whether the algorithm can accurately predict data that were not included in the training process but still fall within the range of the training data. This procedure is standard practice in machine learning applications for droplet microfluidics.^33–36^ We observed an excellent correlation for the holdout data, with *R*^2^ = 0.71 for data where HA (the non-Newtonian phase) was in the dispersed phase, and *R*^2^ = 0.82 for data where HA was in the continuous phase.

Next, we tested our hybrid machine-learning architecture on a new set of data, involving fluids with viscosity curves that differed from those used during training. For the HA in the dispersed phase, we evaluated the network's performance on two HA concentrations: a shear-thinning 0.178 wt% and a negligible shear-thinning 0.0178 wt% (Fig. 5b), with their viscosity curves lying between those used for training. In other words, this test was an interpolation of viscosity curves, but these specific curves had never been seen by the hybrid machine-learning architecture during training. For the 0.0178 wt% data, we observed a strong correlation with *R*^2^ = 0.80 across the entire dataset (Fig. 5c). Interestingly, the data for *q* = 1 deviated more significantly from the predictions. We speculate that this deviation is due to an insufficient number of training data points at *q* = 1, where the droplet typically occupies the entire cross-section of the downstream area of the device. In previous studies,^33–36^ it was consistently observed that the droplet size is generally smaller than the width of the downstream area. For the 0.178 wt% data, the correlation was *R*^2^ = 0.46, primarily due to discrepancies in data at *q* > 0.3 (Fig. 5c). We believe that this limitation is related to the scarcity of data for viscosity curves exhibiting shear-thinning behavior. At such relatively high concentrations of HA, we encountered numerous instabilities in droplet formation (Fig. 2), particularly when *Q*_c_ was comparable to *Q*_d_. Nevertheless, our network was able to deliver relatively accurate predictions for smaller droplets, which were generated at lower values of *q*.

We then repeated the analysis for the configuration where the continuous phase was an unseen non-Newtonian fluid. For this, we used an aqueous polyethylene oxide (PEO) solution with a molecular weight of 4 MDa at 0.4 wt%. This viscosity curve represented an extrapolation beyond those used during training (Fig. 1d), as its zero-shear viscosity was lower than that of any HA solution used for training. We first assessed the accuracy of the hybrid machine-learning architecture in the 190 μm device used during training and observed a correlation of *R*^2^ = 0.83. This result is surprisingly good, given that the viscosity curve differed significantly from those in the training dataset. We further tested the network's accuracy using a different channel with an etching depth of 100 μm. This approach is justified by the fact that our hybrid machine-learning architecture incorporates dimensional analysis, which should enable it to describe different geometries and operating conditions. We observed a correlation of *R*^2^ = 0.48, with better accuracy at lower values of *q*, consistent with the observations described earlier. We also speculate that incorporating the channel height *h* into the training could enhance the quality of the predictions. Although *h* is implicitly represented in a series of dimensionless parameters (such as those involving *U* and *γ̇*), we cannot discount the possibility that treating *h* as an independent training parameter might lead to improved model predictions. Future research should aim to clarify this aspect.

Our approach is also expected to be superior to the use of simple empirical models. Empirical models typically focus on a limited number of variables and may not account for the interaction between a broader set of parameters, such as fluid properties (*e.g.*, shear-dependent viscosity, relaxation time, interfacial tension) and operational conditions (*e.g.*, flow rate and geometry). Neural networks, on the other hand, can effectively handle high-dimensional inputs, learning the complex relationships between multiple parameters and their combined influence on the output, an approach that is unmanageable with traditional empirical methods. This capability is particularly relevant when considering the failure of the master curve, as it suggests that the traditional dimensional analysis used may not have captured the full complexity of the system.

Taken together, the hybrid machine-learning architecture, which combines a neural network followed by a random forest applied to a set of dimensionless parameters, was able to provide accurate predictions regardless of the viscosity curve for both cases of a non-Newtonian dispersed phase and a non-Newtonian continuous phase. Additionally, we found that our network could predict with good accuracy the experimental conditions required to generate droplets in a device having an etching depth of 100 μm, different from the one provided during the training.

## Conclusions

In this study, we addressed the limitations of traditional dimensional analysis in capturing the complex relationships governing non-Newtonian droplet formation in microfluidic systems. Our development of a hybrid machine-learning architecture, which integrates dimensional analysis, enabled accurate predictions of flow rates *Q*_d_ and *Q*_c_ for specified droplet length *L*, height *H*, and the viscosity curves of non-Newtonian phases. This approach effectively combined the strengths of machine learning and dimensional analysis, overcoming challenges that arise from shear-rate dependent viscosities and the variability of flow conditions. Unlike previous work that applied dimensional analysis with machine learning to Newtonian fluids,^51^ our method extends these capabilities to non-Newtonian fluids, making it more versatile and robust. We validated our model through rigorous testing. For holdout data representing 20% of the experimental dataset, the hybrid machine-learning demonstrated excellent predictive accuracy, achieving *R*^2^ = 0.71 for scenarios with HA as the dispersed phase and *R*^2^ = 0.82 for HA in the continuous phase. Additionally, when applied to new datasets with different viscosity curves not included in the training set, the model showed strong interpolation capabilities. For instance, with a shear-thinning HA concentration of 0.0178 wt%, we achieved an *R*^2^ = 0.80. However, predictions for higher concentrations like 0.178 wt% were less accurate (*R*^2^ = 0.46), likely due to the limited data for strongly shear-thinning fluids and the presence of droplet formation instabilities. Even when tested with a new channel with an etching depth of 100 μm, the hybrid network delivered reasonable predictions (*R*^2^ = 0.48), especially at lower values of *q*. Overall, our hybrid machine-learning provides a novel solution that can accurately predict the flow conditions required for generating droplets with various fluid properties. This versatility sets our model apart from previous approaches that either required more extensive knowledge of dimensionless numbers^51^ or were restricted to Newtonian systems.^33,34^ Our findings suggest that integrating machine learning with dimensional analysis is a powerful approach to tackle complex fluid dynamics problems, opening avenues for more efficient and precise control of droplet generation in microfluidic applications.

## Author contributions

(FH) data curation, visualization, writing – review and editing; (CB) conceptualization, formal analysis, methodology, supervision, software, writing – review and editing; (AF) supervision, funding acquisition, writing – review and editing; (SC) supervision, funding acquisition, writing – review and editing; (FDG) conceptualisation, data curation, formal analysis, funding acquisition, investigation, methodology, project administration, resources, supervision, validation, visualisation, writing – original draft, writing – review and editing.

## Conflicts of interest

There are no conflicts to declare.

## Supplementary Material

LC-025-D4LC00946K-s001

LC-025-D4LC00946K-s002

LC-025-D4LC00946K-s003

LC-025-D4LC00946K-s004

LC-025-D4LC00946K-s005

LC-025-D4LC00946K-s006

LC-025-D4LC00946K-s007

LC-025-D4LC00946K-s008

## Data Availability

The data supporting this article have been included as part of the ESI.†
