# Supplementary material for: Dimensional analysis meets AI for non-Newtonian droplet generation
Source: Lab Chip. 2025 Feb 12;25(7):1681–93. doi: 10.1039/d4lc00946k (PMC11834948; doi:10.1039/d4lc00946k)
Supplement: LC-025-D4LC00946K-s006 [file LC-025-D4LC00946K-s006.pdf]

Supplementary Information (SI) for Lab on a Chip.
This journal is © The Royal Society of Chemistry 2025

```
# -*- coding: utf-8 -*-
"""
Created on Wed Sep 25 11:06:13 2024

@author: farnoosh
"""

# Import necessary libraries
import numpy as np
import pandas as pd
import matplotlib.pyplot as plt
from sklearn.preprocessing import MinMaxScaler
from sklearn.metrics import r2_score
from tensorflow.keras.models import Sequential
from tensorflow.keras.layers import Dense
import random
from tensorflow.keras.models import load_model




# Load input data
# Read the input and output CSV files
X = pd.read_csv('/CSV_InputwithZeroes_crossfitParam_10params.csv')
y = pd.read_csv('/CSV_YYs_withZEROES.csv')

# Handle missing data by dropping rows with missing values (blanks)
X = X.dropna()
X_temporary = X.iloc[:, 0:4]
X_cross = X.iloc[:, 4:6]
```

```
ymain = y.loc[X.index]  # Keep only the rows corresponding to non-missing input data

# Extract only the 3rd column (q = Qd/Qc) from the output data
y = ymain.iloc[:, 1].values  # 2 because it's the third column (Python is 0-indexed)
y2 = ymain.iloc[:, 0:2].values

# Extract the values for X
X = X.values
X_temporary = X_temporary.values
X_cross =  X_cross.values

# Scale input data to range [0, 1]
#scaler = MinMaxScaler()
#X_scaled = scaler.fit_transform(X)

# Create a list of all indices
exampleList = list(range(len(X)))

# Define the function to sample the list
def sample_list(example_list, sample_size):
    sampled_list = random.sample(example_list, sample_size)
    for i in sampled_list:
        example_list.remove(i)
    return sampled_list

# Sample the lists as per the requirements
sampled_list1 = sample_list(exampleList, 108)
sampled_list2 = sample_list(exampleList, 108)
```

```
sampled_list3 = sample_list(exampleList, 108)

sampled_list4 = sample_list(exampleList, 108)

sampled_list5 = sample_list(exampleList, 108)

sampled_list6 = sample_list(exampleList, 108)

sampled_list7 = sample_list(exampleList, 108)

sampled_list8 = sample_list(exampleList, 108)

sampled_list9 = sample_list(exampleList, 108)

sampled_list10 = sample_list(exampleList, 107)


# Combine the sampled lists for training and validation

FINAL = sampled_list1 +sampled_list2 + sampled_list3 + sampled_list4 + sampled_list5 +
sampled_list7 + sampled_list8 + sampled_list9 + sampled_list10

#FINAL2 = sampled_list10

FINAL2 = sampled_list6 + sampled_list8




# Prepare training and validation datasets

X_train = X[FINAL]

X_cross_train = X_cross[FINAL]

X_cross_val = X_cross[FINAL2]

y_train = y[FINAL]

X_val = X[FINAL2]

y_val = y[FINAL2]



Ytrainpart2 = y2[FINAL,:]

Yvalpart2 = y2[FINAL2,:]
```

```
# Create the Neural Network model
model = Sequential()
model.add(Dense(500, input_dim=X_train.shape[1], activation='relu'))
model.add(Dense(300, activation='relu'))
model.add(Dense(100, activation='relu'))
model.add(Dense(1, activation='linear'))  # Output layer for regression (predicting q =
Qd/Qc)

# Compile the model
model.compile(loss='mean_squared_error', optimizer='adam',
metrics=['mean_squared_error'])

# Train the model
history = model.fit(X_train, y_train, epochs=1000, batch_size=10, validation_data=(X_val,
y_val), verbose=1)

# Predict on validation set
y_pred = model.predict(X_val)
y_predpart2 = model.predict(X_train)

# Calculate R^2 score
r2 = r2_score(y_val, y_pred)

# Plot Q experimental (Qd/Qc) vs Q predicted (Qd/Qc)
plt.figure(figsize=(8, 6))
plt.scatter(y_val, y_pred, color='blue', label='Data points')
plt.plot([min(y_val), max(y_val)], [min(y_val), max(y_val)], color='red', linewidth=2,
label='Perfect Prediction Line')
```

```
plt.xlabel('Qc Experimental')

plt.ylabel('Qc Predicted ')

plt.title(f'Qc Experimental Predicted (R² = {r2:.3f})')

plt.legend()

plt.show()


#

AA = np.mean(np.abs([y_predpart2-y_train]))



#model.save('C:/Users/farno/OneDrive/Desktop/Secondment/presentations/Claire_ML/26sep/modelQc.h5')

#model = load_model('C:/Users/farno/OneDrive/Desktop/Secondment/presentations/Claire_ML/26sep/modelQcc.h5')



ypredtest_flow2 = model.predict(X_val)

ypredtest_flow3 = model.predict(X_train)



X3_min = ypredtest_flow3 - 40

X3_max = ypredtest_flow3 + 40

X3_mid = (X3_min + X3_max) / 2  # midpoint as a representative


X3val_min = ypredtest_flow2 - 40

X3val_max = ypredtest_flow2 + 40

X3val_mid = (X3val_min + X3val_max) / 2  # midpoint as a representative
```

```
# Calculate U_c, shear_rate, eta_c, Wi, alfa, Ca_c, Re_c using Qc_pred
depth_of_channel = 190 / 1000  # 190 micrometers converted to meters
cross_section_area = (190 / 1000) * (195 / 1000) / 60  # (190µm x 195µm) converted to
m²/60

U_c = y_pred / cross_section_area  # Continuous phase velocity
shear_rate = U_c / depth_of_channel  # Shear rate

# Calculate eta_c (avoid division by zero by adding a small epsilon)
epsilon = 1e-10
eta_c = X_cross_val[:, 0] / ((1 + X_val[:, 0] * shear_rate.flatten()) ** X_cross_val[:, 1] +
epsilon)

# Calculate Wi, alfa, Ca_c, Re_c (handle potential divisions by zero or near-zero values)
Wi = X_val[:, 3] * shear_rate.flatten()
alfa = 0.029 / (eta_c . flatten() + epsilon)
Ca_c = (eta_c. flatten() * U_c. flatten() / 1000) / (X_val[:, 2] / 1000 + epsilon)# 2 replaced to
y_predpart2
Re_c = (1000 * U_c. flatten() / 1000) * (190 / 10 ** 6) / (eta_c. flatten() + epsilon)

X3 = ypredtest_flow2
X4 = alfa
X5 = Wi
X6 = Re_c
```

```
X7 = Ca_c

X_val_new = np.hstack([X_val, X7.flatten().reshape(-1, 1),
            X3.reshape(-1, 1),
            X4.reshape(-1, 1),
            X5.reshape(-1, 1),
            X6.reshape(-1, 1)])


U_c_val = ypredtest_flow2 / cross_section_area
U_c_train = ypredtest_flow3 / cross_section_area
shear_rate_val = U_c_val / depth_of_channel
shear_rate_train = U_c_train / depth_of_channel # Shear rate
eta_c2 = X_cross_train[:, 0] / ((1 + X_train[:, 3] * shear_rate_train.flatten()) ** X_cross_train[:, 1] + epsilon)

# Calculate Wi, alfa, Ca_c, Re_c (handle potential divisions by zero or near-zero values)
Wi2 = X_train[:, 3] * shear_rate_train.flatten()
alfa2 = 0.029 / (eta_c2.flatten() + epsilon)
Ca_c2 = (eta_c2.flatten() * U_c_train.flatten() / 1000) / (X_train[:, 2] / 1000 + epsilon)# 2 replaced to y_predpart2
Re_c2 = (1000 * U_c_train.flatten() / 1000) * (190 / 10 ** 6) / (eta_c2.flatten() + epsilon)


X3_2 = ypredtest_flow3
X4_2 = alfa2
X5_2 = Wi2
X6_2 = Re_c2
```

```
X7_2 = Ca_c2

X_train_new = np.hstack([X_train, X7_2.flatten().reshape(-1, 1),
            X3_2.reshape(-1, 1),
            X4_2.reshape(-1, 1),
            X5_2.reshape(-1, 1),
            X6_2.reshape(-1, 1)])

from sklearn.ensemble import RandomForestRegressor

# Initialize Random Forest Regressor
rf = RandomForestRegressor(n_estimators=500, random_state=42)

# Train the model on your dataset
rf.fit(X_train_new, Ytrainpart2)

# Predict on new data

y_pred = rf.predict(X_val_new)
plt.figure(figsize=(8, 8))
plt.scatter( Yvalpart2[:,0]/Yvalpart2[:,1],y_pred[:,0]/y_pred[:,1], c='blue', label='Predicted vs. Exp')
```

```
# Values you want to save
Yval0 = Yvalpart2[:, 0]
Yval1 = Yvalpart2[:, 1]
y_pred0 = y_pred[:, 0]
y_pred1 = y_pred[:, 1]
Yval_ratio = Yval0 / Yval1
y_pred_ratio = y_pred0 / y_pred1




####################################################Test
sets##############################################################


##################################peo test 190
channel#######################################


X = pd.read_csv('/CSV_InputwithZeroes_peo.csv')
y = pd.read_csv('/CSV_YYs_withZEROES_peo.csv')


# Handle missing data by dropping rows with missing values (blanks)
X = X.dropna()
X_temporary = X.iloc[:, 0:4]
X_cross = X.iloc[:, 4:6]


ymain = y.loc[X.index]  # Keep only the rows corresponding to non-missing input data
```

```
#Extract only the 3rd column (q = Qd/Qc) from the output data
y = ymain.iloc[:, 1].values  # 2 because it's the third column (Python is 0-indexed)
y2 = ymain.iloc[:, 0:2].values

# Extract the values for X
X = X.values
X_temporary = X_temporary.values
X_cross =  X_cross.values



# Prepare training and validation datasets
X_test_peo = X
X_cross_test_peo = X_cross
y_test_peo = y

y_test_part2 = y2

y_predtest_flow3 = model.predict(X_test_peo)



X3_min = y_predtest_flow3 - 40
X3_max = y_predtest_flow3 + 40
X3_mid = (X3_min + X3_max) / 2  # midpoint as a representative


# Calculate U_c, shear_rate, eta_c, Wi, alfa, Ca_c, Re_c using Qc_pred
```

```
depth_of_channel = 190 / 1000  # 190 micrometers converted to meters

cross_section_area = (190 / 1000) * (195 / 1000) / 60  # (190µm x 195µm) converted to m²/60


U_c = y_predtest_flow3 / cross_section_area  # Continuous phase velocity

shear_rate = U_c / depth_of_channel  # Shear rate


# Calculate eta_c (avoid division by zero by adding a small epsilon)

epsilon = 1e-10


eta_c = X_cross_test_peo[:, 0] / ((1 + X_test_peo[:, 0] * shear_rate.flatten()) ** X_cross_test_peo[:, 1] + epsilon)


# Calculate Wi, alfa, Ca_c, Re_c (handle potential divisions by zero or near-zero values)

Wi = X_test_peo[:, 3] * shear_rate.flatten()

alfa = 0.029 / (eta_c . flatten() + epsilon)

Ca_c = (eta_c. flatten() * U_c. flatten() / 1000) / (X_test_peo[:, 2] / 1000 + epsilon)# 2 replaced to y_predpart2

Re_c = (1000 * U_c. flatten() / 1000) * (190 / 10 ** 6) / (eta_c. flatten() + epsilon)


X3 = y_predtest_flow3

X4 = alfa

X5 = Wi

X6 = Re_c

X7 = Ca_c
```

```
X_test_new = np.hstack([X_test_peo, X7.flatten().reshape(-1, 1),
            X3.reshape(-1, 1),
            X4.reshape(-1, 1),
            X5.reshape(-1, 1),
            X6.reshape(-1, 1)])


y_pred = rf.predict(X_test_new)


plt.figure(figsize=(8, 8))
plt.scatter( y_test_part2[:,0]/y_test_part2[:,1],y_pred[:,0]/y_pred[:,1], c='blue',
label='Predicted vs. Exp')


Yval0 = y_test_part2[:, 0]
Yval1 = y_test_part2[:, 1]
y_pred0 = y_pred[:, 0]
y_pred1 = y_pred[:, 1]
Yval_ratio = Yval0 / Yval1
y_pred_ratio = y_pred0 / y_pred1




####################################################peo test 100
channel################
X = pd.read_csv('/CSV_InputwithZeroes_geometry100_peo.csv')
y = pd.read_csv('/CSV_YYs_withZEROES_geometry100_peo.csv')


# Handle missing data by dropping rows with missing values (blanks)
```

```
X = X.dropna()
X_temporary = X.iloc[:, 0:4]
X_cross = X.iloc[:, 4:6]

ymain = y.loc[X.index]  # Keep only the rows corresponding to non-missing input data

#Extract only the 3rd column (q = Qd/Qc) from the output data
y = ymain.iloc[:, 1].values  # 2 because it's the third column (Python is 0-indexed)
y2 = ymain.iloc[:, 0:2].values

# Extract the values for X
X = X.values
X_temporary = X_temporary.values
X_cross =  X_cross.values



# Prepare training and validation datasets
X_test_peo = X
X_cross_test_peo = X_cross
y_test_peo = y

y_test_part2 = y2

y_predtest_flow3 = model.predict(X_test_peo)



X3_min = y_predtest_flow3 - 40
```

```
X3_max = y_predtest_flow3 + 40

X3_mid = (X3_min + X3_max) / 2  # midpoint as a representative



# Calculate U_c, shear_rate, eta_c, Wi, alfa, Ca_c, Re_c using Qc_pred

depth_of_channel = 100 / 1000  # 190 micrometers converted to meters

cross_section_area = (100 / 1000) * (105 / 1000) / 60  # (190µm x 195µm) converted to m²/60


U_c = y_predtest_flow3 / cross_section_area  # Continuous phase velocity

shear_rate = U_c / depth_of_channel  # Shear rate


# Calculate eta_c (avoid division by zero by adding a small epsilon)

epsilon = 1e-10


eta_c = X_cross_test_peo[:, 0] / ((1 + X_test_peo[:, 0] * shear_rate.flatten()) ** X_cross_test_peo[:, 1] + epsilon)

#



# Calculate Wi, alfa, Ca_c, Re_c (handle potential divisions by zero or near-zero values)

Wi = X_test_peo[:, 3] * shear_rate.flatten()

alfa = 0.029 / (eta_c . flatten() + epsilon)

Ca_c = (eta_c. flatten() * U_c. flatten() / 1000) / (X_test_peo[:, 2] / 1000 + epsilon)# 2 replaced to y_predpart2

Re_c = (1000 * U_c. flatten() / 1000) * (100 / 10 ** 6) / (eta_c. flatten() + epsilon)
```

```
X3 = y_predtest_flow3

X4 = alfa

X5 = Wi

X6 = Re_c

X7 = Ca_c


X_test_new = np.hstack([X_test_peo, X7.flatten().reshape(-1, 1),

            X3.reshape(-1, 1),

            X4.reshape(-1, 1),

            X5.reshape(-1, 1),

            X6.reshape(-1, 1)])



y_pred = rf.predict(X_test_new)

plt.figure(figsize=(8, 8))

plt.scatter( y_test_part2[:,0]/y_test_part2[:,1],y_pred[:,0]/y_pred[:,1], c='blue',
label='Predicted vs. Exp')



Yval00 = y_test_part2[:, 0]

Yval11 = y_test_part2[:, 1]

y_pred0 = y_pred[:, 0]

y_pred1 = y_pred[:, 1]

Yval_ratio = Yval00 / Yval11

y_pred_ratio = y_pred0 / y_pred1
```

```
###################################################Geometry 100# HA
################

X = pd.read_csv('/CSV_InputwithZeroes_geometry_HA100_CROSSparam.csv')

y = pd.read_csv('/CSV_YYs_withZEROES_geometry_HA100.csv')


# Handle missing data by dropping rows with missing values (blanks)

X = X.dropna()

X_temporary = X.iloc[:, 0:4]

X_cross = X.iloc[:, 4:6]


ymain = y.loc[X.index]  # Keep only the rows corresponding to non-missing input data


#Extract only the 3rd column (q = Qd/Qc) from the output data

y = ymain.iloc[:, 1].values  # 2 because it's the third column (Python is 0-indexed)

y2 = ymain.iloc[:, 0:2].values


# Extract the values for X

X = X.values

X_temporary = X_temporary.values

X_cross =  X_cross.values




# Prepare training and validation datasets
```

```
X_test_peo = X
X_cross_test_peo = X_cross
y_test_peo = y

y_test_part2 = y2

y_predtest_flow3 = model.predict(X_test_peo)



X3_min = y_predtest_flow3 - 40
X3_max = y_predtest_flow3 + 40
X3_mid = (X3_min + X3_max) / 2  # midpoint as a representative



# Calculate U_c, shear_rate, eta_c, Wi, alfa, Ca_c, Re_c using Qc_pred
depth_of_channel = 100 / 1000  # 190 micrometers converted to meters
cross_section_area = (100 / 1000) * (105 / 1000) / 60  # (190µm x 195µm) converted to m²/60

U_c = y_predtest_flow3 / cross_section_area  # Continuous phase velocity
shear_rate = U_c / depth_of_channel  # Shear rate

# Calculate eta_c (avoid division by zero by adding a small epsilon)
epsilon = 1e-10

eta_c = X_cross_test_peo[:, 0] / ((1 + X_test_peo[:, 0] * shear_rate.flatten()) ** X_cross_test_peo[:, 1] + epsilon)
#
```

```
# Calculate Wi, alfa, Ca_c, Re_c (handle potential divisions by zero or near-zero values)
Wi = X_test_peo[:, 3] * shear_rate.flatten()
alfa = 0.029 / (eta_c . flatten() + epsilon)
Ca_c = (eta_c. flatten() * U_c. flatten() / 1000) / (X_test_peo[:, 2] / 1000 + epsilon)# 2 replaced to y_predpart2
Re_c = (1000 * U_c. flatten() / 1000) * (100 / 10 ** 6) / (eta_c. flatten() + epsilon)


X3 = y_predtest_flow3
X4 = alfa
X5 = Wi
X6 = Re_c
X7 = Ca_c

X_test_new = np.hstack([X_test_peo, X7.flatten().reshape(-1, 1),
        X3.reshape(-1, 1),
        X4.reshape(-1, 1),
        X5.reshape(-1, 1),
        X6.reshape(-1, 1)])


y_pred = rf.predict(X_test_new)
plt.figure(figsize=(8, 8))
plt.scatter( y_test_part2[:,0]/y_test_part2[:,1],y_pred[:,0]/y_pred[:,1], c='blue', label='Predicted vs. Exp')
```

```
Yval00 = y_test_part2[:, 0]

Yval11 = y_test_part2[:, 1]

y_pred0 = y_pred[:, 0]

y_pred1 = y_pred[:, 1]

Yval_ratio = Yval00 / Yval11

y_pred_ratio = y_pred0 / y_pred1
```
