# Supplementary material for: Dimensional analysis meets AI for non-Newtonian droplet generation
Source: Lab Chip. 2025 Feb 12;25(7):1681–93. doi: 10.1039/d4lc00946k (PMC11834948; doi:10.1039/d4lc00946k)
Supplement: LC-025-D4LC00946K-s007 [file LC-025-D4LC00946K-s007.pdf]

Supplementary Information (SI) for Lab on a Chip.
This journal is © The Royal Society of Chemistry 2025

```
# File paths for convenience
data_file = 'C:/Users/Paul/Downloads/reduced_data_newer2.xlsx'
y_values_file = 'C:/Users/Paul/Downloads/Reduced_yvalues.xlsx'
# Function to load and clean data from a specific sheet
def load_and_clean_data(file, sheet_name):
    df = pd.read_excel(file, sheet_name=sheet_name).values
    df = df[~np.isnan(df).any(axis=1)]
    # Remove rows with NaN values
    Xa = df[:, [0, 1, 2, 3]]
    Xb = df[:, [7, 8]]
    mask = ~((Xa[:, 0] == 0) & (Xa[:, 1] == 0))
    return Xa[mask], Xb[mask], df[mask, 4:5], df[mask, 4:6]

# Load data for each sheet and assign to variables
sheets_data = ['HA0pt01', 'HA0pt0316', 'HA0pt1', 'HA0pt316', 'HA0pt562', 'HA1']
X_a_list, X_b_list, Y_list, Y_b_list = [], [], [], []
for sheet in sheets_data:
    Xa, Xb, Y, Yb = load_and_clean_data(data_file, sheet)
     X_a_list.append(Xa)
     X_b_list.append(Xb)
    Y_list.append(Y)
    Y_b_list.append(Yb)

Xtemp = np.vstack(X_a_list)
Ytemp = np.concatenate(Y_list)
Xadditional = np.vstack(X_b_list)
Ypart2 = np.concatenate(Y_b_list)


D = np.shape(Xtemp)
total_samples = 12
```

```
extra_samples = 14 # Create a list of indices based on the shape of Xtemp

exampleList = list(range(D[0])) # Function to sample and remove elements from exampleList

def sample_and_remove(num_samples):

    sampled_list = random.sample(exampleList, num_samples)

    for item in sampled_list:

        exampleList.remove(item)

        return sampled_list

# Sample lists

sampled_lists = [sample_and_remove(total_samples) for _ in range(9)]

sampled_list10 = sample_and_remove(extra_samples)

# Construct FINAL and FINAL2

FINAL = ( sampled_lists[0] + sampled_lists[1] + sampled_lists[2] + sampled_lists[3] + sampled_lists[4]
+ sampled_lists[5] + sampled_lists[6] + sampled_lists[7] + sampled_lists[8] )

FINAL2 = sampled_list10 + sampled_lists[1]




Xtrain = Xtemp[FINAL,:]

Ytrain = Ytemp[FINAL]

Ytrainpart2 = Ypart2[FINAL,:]

Xtrain_parm = Xadditional[FINAL,:]

Xval = Xtemp[FINAL2,:]

Xval_parm = Xadditional[FINAL2,:]

Yval = Ytemp[FINAL2]

Yvalpart2 = Ypart2[FINAL2,:]

#Define model

input_layer = Input(shape=(4,))

x = Dense(600, activation='relu')(input_layer)

x = Dense(300, activation='relu')(x)

x = Dense(200, activation='relu')(x)

output_layer = Dense(1, activation='linear')(x)
```

```
model1 = Model(input_layer, output_layer)
model1.compile(loss='mae', optimizer='adam', metrics=['accuracy'])

# Train the first model
history1 = model1.fit(Xtrain, Ytrain, epochs=6000, batch_size=10, verbose=2, validation_split=0.1)



ypredtest_flow2 = model1.predict(Xtrain)
AA = np.mean(np.abs([ypredtest_flow2-Ytrain]))
ypredtest_flow3 = model1.predict(Xval)

X3_mid = ypredtest_flow2
X3val_mid = ypredtest_flow3;

# Calculate U_c, shear_rate, eta_c, Wi, alfa, Ca_c, Re_c using Qc_pred
depth_of_channel = 100 / 1000  # 100 micrometers converted to meters
cross_section_area = (100 / 1000) * (105 / 1000) / 60  # (100µm x 105µm) converted to m²/60

U_d = ypredtest_flow2/ cross_section_area  #  phase velocity
shear_rate = U_d / depth_of_channel  # Shear rate
errorSR = 3/cross_section_area /depth_of_channel

U_dval = ypredtest_flow3/ cross_section_area  #  phase velocity
shear_rateval = U_dval / depth_of_channel  # Shear rate
errorSRval = 3/cross_section_area /depth_of_channel




# Calculate eta_c (avoid division by zero by adding a small epsilon)
epsilon = 1e-10
```

```
eta_d = Xtrain_parm[:, 0] / (((1 + Xtrain[:,3] * shear_rate.flatten()) ** Xtrain_parm[:, 1] + epsilon))
epsilon = 1e-10
eta_dval = Xval_parm[:, 0] / (((1 + Xval[:,3] * shear_rateval.flatten()) ** Xval_parm[:, 1] + epsilon))
# Calculate Wi, alfa, Ca_c, Re_c (handle potential divisions by zero or near-zero values)
#touse
Wi = Xtrain[:,3].reshape(-1, 1) * shear_rate
alfa = (eta_d)/0.29
Wival = Xval[:, 3].reshape(-1, 1)  * shear_rateval
alfaval = (eta_dval)/0.29
Ca_d = (eta_d * U_d.flatten() / 1000) / ((Xtrain[:, 2] / 1000) + epsilon)
Re_d = (1000 * U_d.flatten() / 1000) * (100 / 10 ** 6) / (eta_d + epsilon)


Ca_dval = (eta_dval * U_dval.flatten() / 1000) / ((Xval[:, 2] / 1000) + epsilon)
Re_dval = (1000 * U_dval.flatten() / 1000) * (100 / 10 ** 6) / (eta_dval + epsilon)


X4_mid = Wi
X5_mid = Re_d
X6_mid = Ca_d
X7_mid = alfa


X4val_mid = Wival
X5val_mid = Re_dval
X6val_mid = Ca_dval
X7val_mid = alfaval

X = np.hstack([Xtrain, X3_mid.flatten().reshape(-1, 1),
        X4_mid.reshape(-1, 1),
        X5_mid.reshape(-1, 1),
```

```
            X6_mid.reshape(-1, 1),
            X7_mid.reshape(-1, 1)])



Xval2 = np.hstack([Xval, X3val_mid.flatten().reshape(-1, 1),
            X4val_mid.reshape(-1, 1),
            X5val_mid.reshape(-1, 1),
            X6val_mid.reshape(-1, 1),
            X7val_mid.reshape(-1, 1)])


from sklearn.ensemble import RandomForestRegressor

# Initialize Random Forest Regressor
rf = RandomForestRegressor(n_estimators=500, random_state=42)

# Train the model on your dataset
rf.fit(X, Ytrainpart2)

# Predict on new data
y_pred = rf.predict(Xval2)
```
