# Supplementary material for: Dimensional analysis meets AI for non-Newtonian droplet generation
Source: Lab Chip. 2025 Feb 12;25(7):1681–93. doi: 10.1039/d4lc00946k (PMC11834948; doi:10.1039/d4lc00946k)
Supplement: LC-025-D4LC00946K-s008 [file LC-025-D4LC00946K-s008.pdf]

Supplementary Information (SI) for Lab on a Chip.
This journal is © The Royal Society of Chemistry 2025

Supplementary Information to Dimensional Analysis Meets AI for non-Newtonian Droplet Generation

Farnoosh Hormozinezhad,[1] Claire Barnes,[2] Alexandre Fabregat,[1] Salvatore Cito,[1] Francesco Del Giudice[3]

[1]Departament d'Enginyeria Mecànica, Universitat Rovira i Virgili, Tarragona, Spain
[2]Department of Biomedical Engineering, Swansea University, UK.
[3]Complex Fluids Research Group, Department of Chemical Engineering, Swansea University, Fabian Way, SA1 8EN, Swansea, UK. E-mail: francesco.delgiudice@swansea.ac.uk

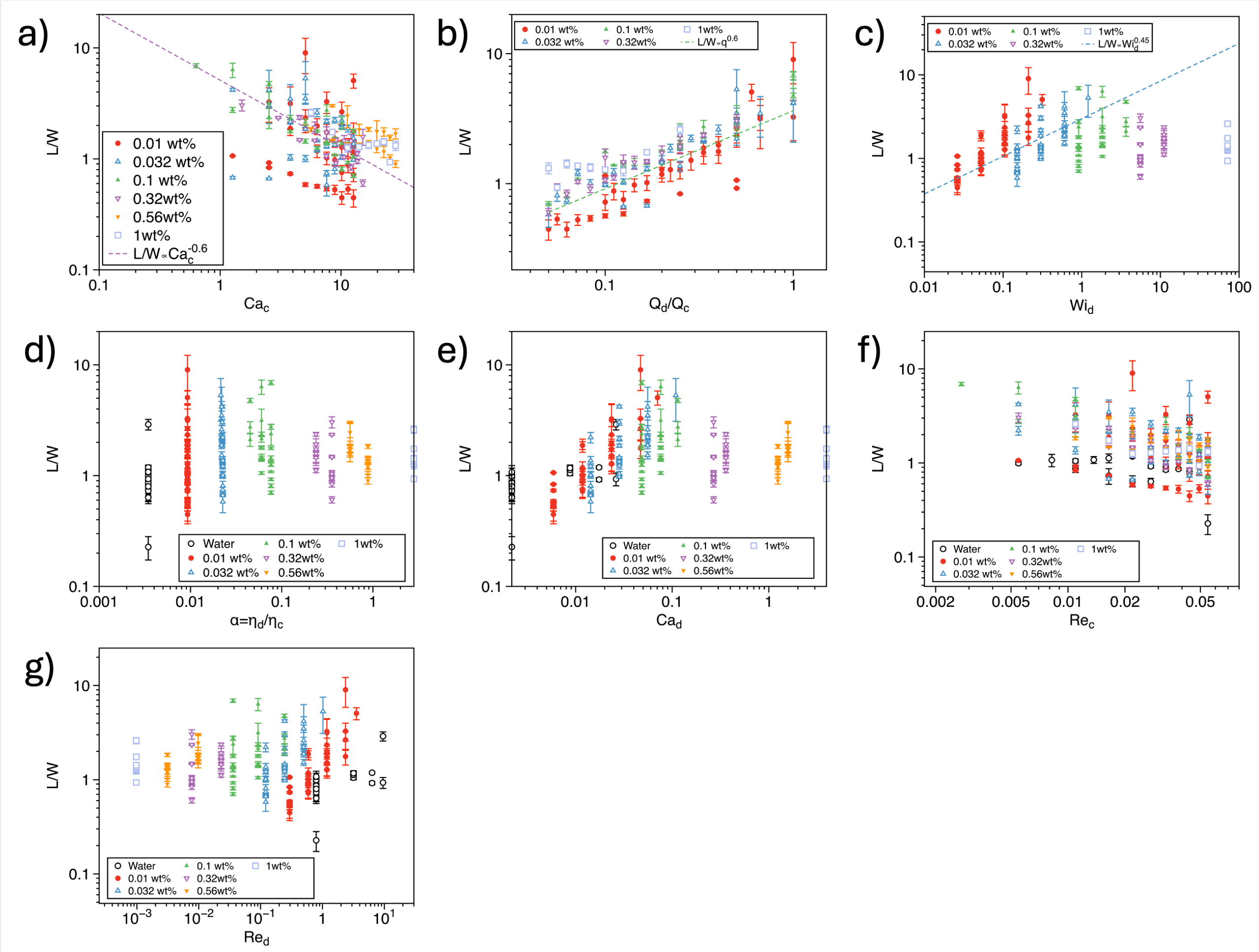

Figure S1: Behaviour of droplet length L normalised y the channel width W as a function of different dimensionless parameters. Dashed lines are the best fit of a subset of the data and are colour-coded to the fitted data. When no fitting is available, we could not identify any clear correlation.

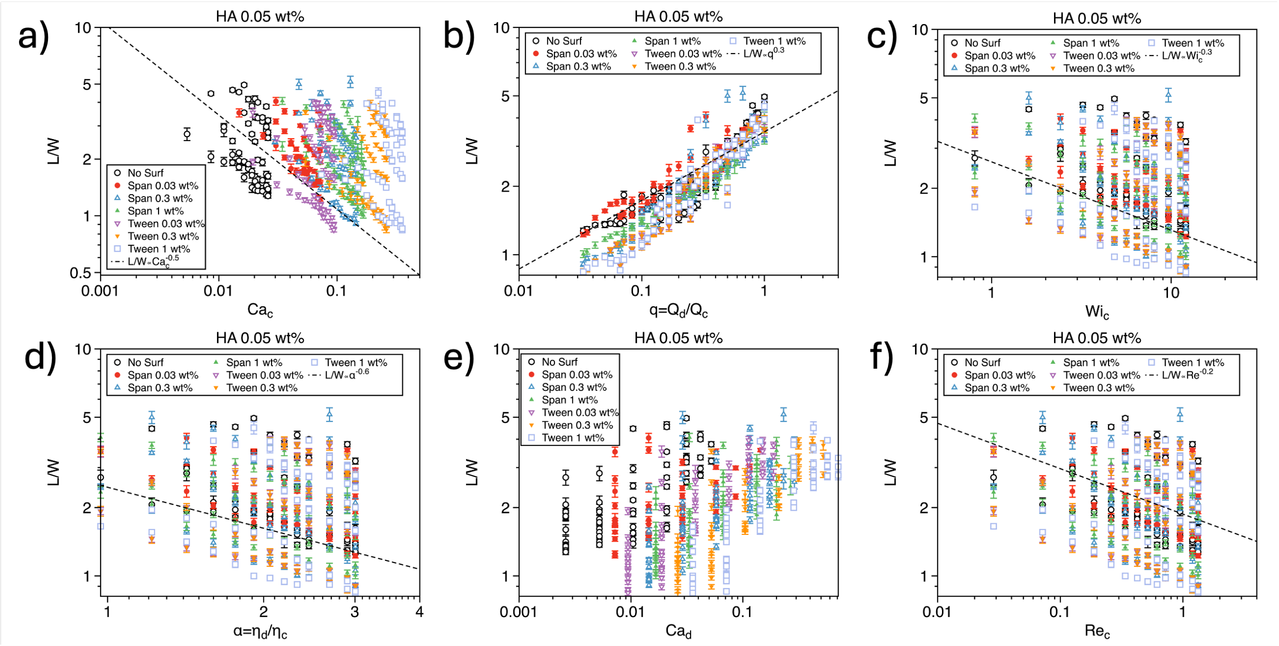

Figure S2: Behaviour of droplet length L normalised y the channel width W as a function of different dimensionless parameters. Dashed lines are the best fit of a subset of the data and are colour-coded to the fitted data. When no fitting is available, we could not identify any clear correlation.

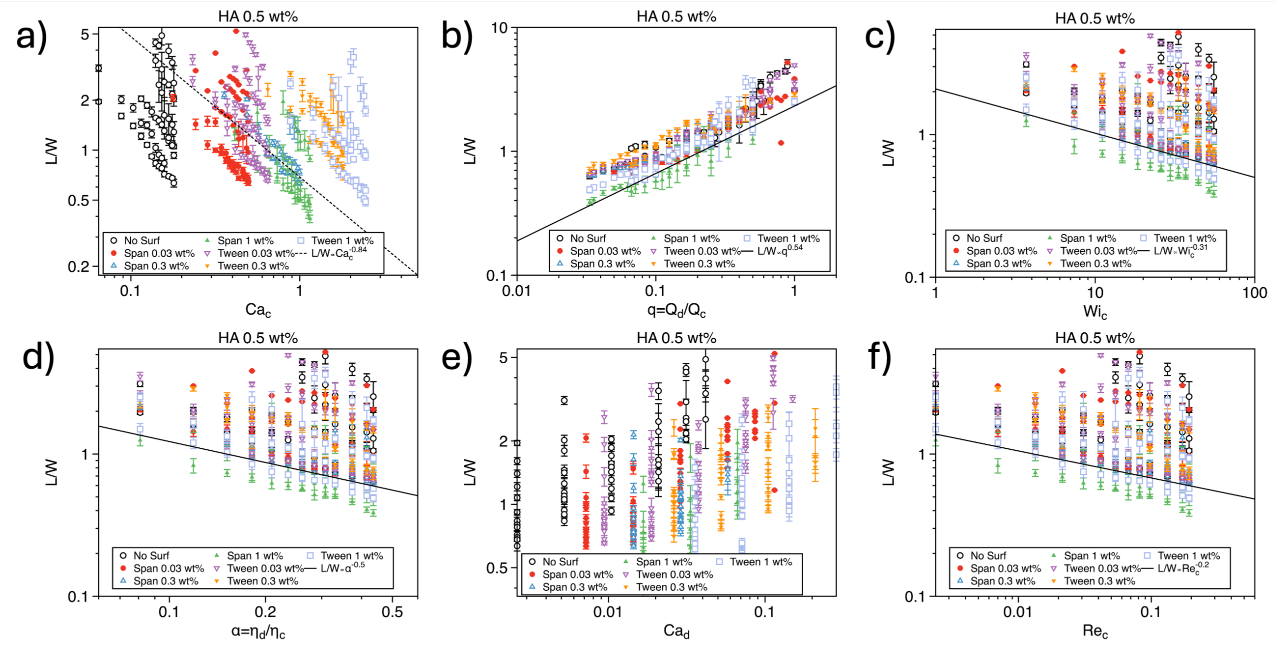

Figure S3: Behaviour of droplet length L normalised y the channel width W as a function of different dimensionless parameters. Dashed lines are the best fit of a subset of the data and are colour-coded to the fitted data. When no fitting is available, we could not identify any clear correlation.
